# Muskrat Island: Behavioral Shifts of an Insular Muskrat ( *Ondatra zibethicus* ) Population in the Gulf of Maine

**DOI:** 10.1002/ece3.71502

**Published:** 2025-06-01

**Authors:** Alexis M. Mychajliw, Max Zeltsar, John Dennis, Maddie E. Ellms, Dylan Titmuss, Kristen M. Covino, Sara Williams, Shrushti Modi, Courtney A. Hofman

**Affiliations:** ^1^ Department of Biology Middlebury College Middlebury Vermont USA; ^2^ Program in Environmental Studies Middlebury College Middlebury Vermont USA; ^3^ Shoals Marine Laboratory Kittery Maine USA; ^4^ Mi'kmaq Nation; ^5^ Department of Applied Physics and Oceanography Woods Hole Oceanographic Institution Woods Hole Massachusetts USA; ^6^ Biology Department Loyola Marymount University Los Angeles California USA; ^7^ Laboratories of Molecular Anthropology & Microbiome Research University of Oklahoma Norman Oklahoma USA; ^8^ Department of Anthropology University of Oklahoma Norman Oklahoma USA; ^9^ Wildlife Institute of India Dehradun Uttarakhand India

**Keywords:** camera trap, furbearer, introduced, island, Maine, mammal

## Abstract

The aftermath of the North American fur trade resulted in the depletion of many furbearing mammal populations in their native North American range while simultaneously creating invasive populations of these species through translocations worldwide. Here, we document the ongoing results of this mass ecological experiment by describing the natural history of a remnant fur colony of muskrats ( *Ondatra zibethicus* ) putatively introduced to the Isles of Shoals archipelago in the Gulf of Maine in the early 20th century. Through a combination of intensive surveys and camera trapping, we document how muskrats have been influenced by insular conditions under expectations of island biogeographic theory. Unlike other translocated muskrats that have produced successful wetland‐restricted populations in continental Europe and Asia, the Shoals muskrats appear to have shifted their habitat use and lodge building behavior and have encountered a new predator: gulls (Laridae). This Nature Note formalizes decades of anecdotal observations and provides important insight into the ecological flexibility of muskrats given the paradox of a species that is apparently now declining in its native range but expanding outside of it.

## Graphical abstract

The North American fur trade fundamentally shifted baselines of furbearing mammals worldwide. Using camera traps and visual surveys, we document unexpected ecological and behavioral characteristics of a population of muskrats ( *Ondatra zibethicus* ) that was likely introduced to the Isles of Shoals (Maine/New Hampshire, USA) in the early 1900s.

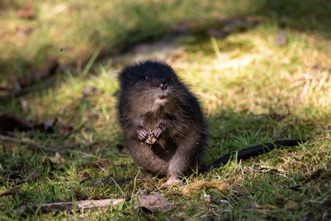

## 1 Introduction

**FIGURE 1:**
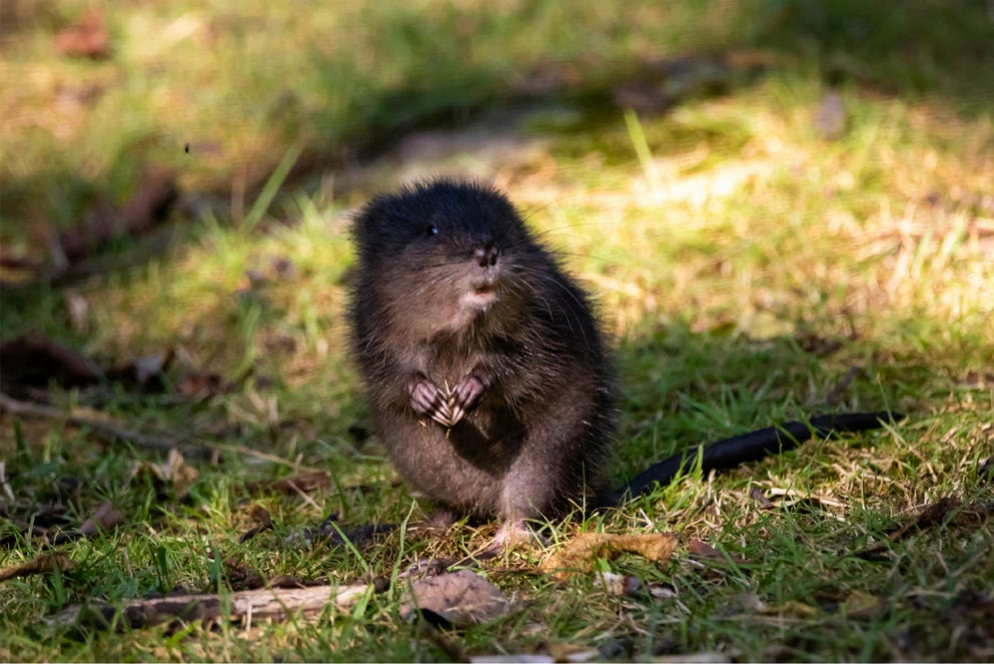
A juvenile muskrat ( *Ondatra zibethicus* ) in its typical insular habitat on Appledore Island, Isles of Shoals, Maine. Photo by M. Zeltsar.

Novel ecological interactions (i.e., those deviating from historical baselines (Guiden et al. 2019)) have become ubiquitous facets of Anthropocene life (Radeloff et al. 2015). Although many of these interactions may be truly novel (e.g., when species have crossed major biogeographic boundaries), some may only appear to be novel due to baseline shifts that precede human memory (Silliman et al. 2018; Jackson et al. 2001; McClenachan et al. 2024). The North American fur trade—beginning in the 1500s and extending through the 1800s CE—widely distorted the baselines of abundance and range of furbearing mammals (species that produce fur of commercial value such as beavers, muskrats, and minks) (White et al. 2015; Mychajliw et al. 2023). Failing to account for this anthropogenic imprint on furbearers can result in an incorrect assessment of species statuses; perceived novel expansions might instead be recoveries (Collins et al. 2020), or perceived native populations may have been translocations (Heter 1950; Storer 1937). Such cryptic range augmentations are effectively “natural experiments” in understanding ecological flexibility and niche delimitation. Here we describe novel ecological interactions resulting from such a natural experiment, a historical introduction of muskrats ( *Ondatra zibethicus* ; Figure 1; ki'kwesu or kiwhos in Wabanaki language (Dennis 2016)) to the Isles of Shoals archipelago within the overall North American native range (Cassola 2016; Mychajliw and Harrison 2014).

Muskrats are semiaquatic microtine rodents that weigh approximately 1 kg and, similar to beavers, are prized for their dense, waterproof underfur (Willner et al. 1980). Muskrats consume the roots and other basal portions of aquatic plants such as cattails (*Typha* spp.), but will opportunistically eat small mollusks, fish, and crayfish (Willner et al. 1980; Edelman et al. 2015; Adams and Rosamond 2022). Typically, muskrats use emergent vegetation and macrophytes (rather than sticks, like beavers) to construct dome‐shaped lodges in which an underwater entrance leads to dry interior rooms (Westworth 1974). A single family of muskrats can have numerous specialized structures for living and feeding within their territory aside from lodges, including bank burrows created by digging into the soft sediment alongside a riparian area and “push up” platforms resulting from pushing plant material up to the surface under ice. Thus, they are considered obligate wetland associates (Willner et al. 1980) and are thought to only inhabit upland areas as marginal habitat due to flooding, drought, or freeze outs (Errington et al. 1963).

Muskrats can profoundly alter the wetland ecosystems they inhabit (Connors et al. 2000; Nyman et al. 1993; Kua et al. 2020). Within their native range, their ecological engineering is largely viewed as positive, providing structures for use by birds, reptiles, and amphibians (Baici et al. 2024; Kiviat 1978; Hickey and Malecki 1997; Litzgus and Brooks 2000). Impacts of such magnitude, however, cause them to be labeled as pests in their invasive range, which spans Europe, Asia, and South America (Danell 1996; Cassola 2016; GISD 2025). Muskrat activity can lead to human‐wildlife conflict when it results in clogged culverts, breached berms, and flooding of human infrastructure, potentially interfering with restoration (Storer 1937; Shuler 2000; Kadlec et al. 2007; Skyrienė and Paulauskas 2012; Crego et al. 2016).

**FIGURE 2:**
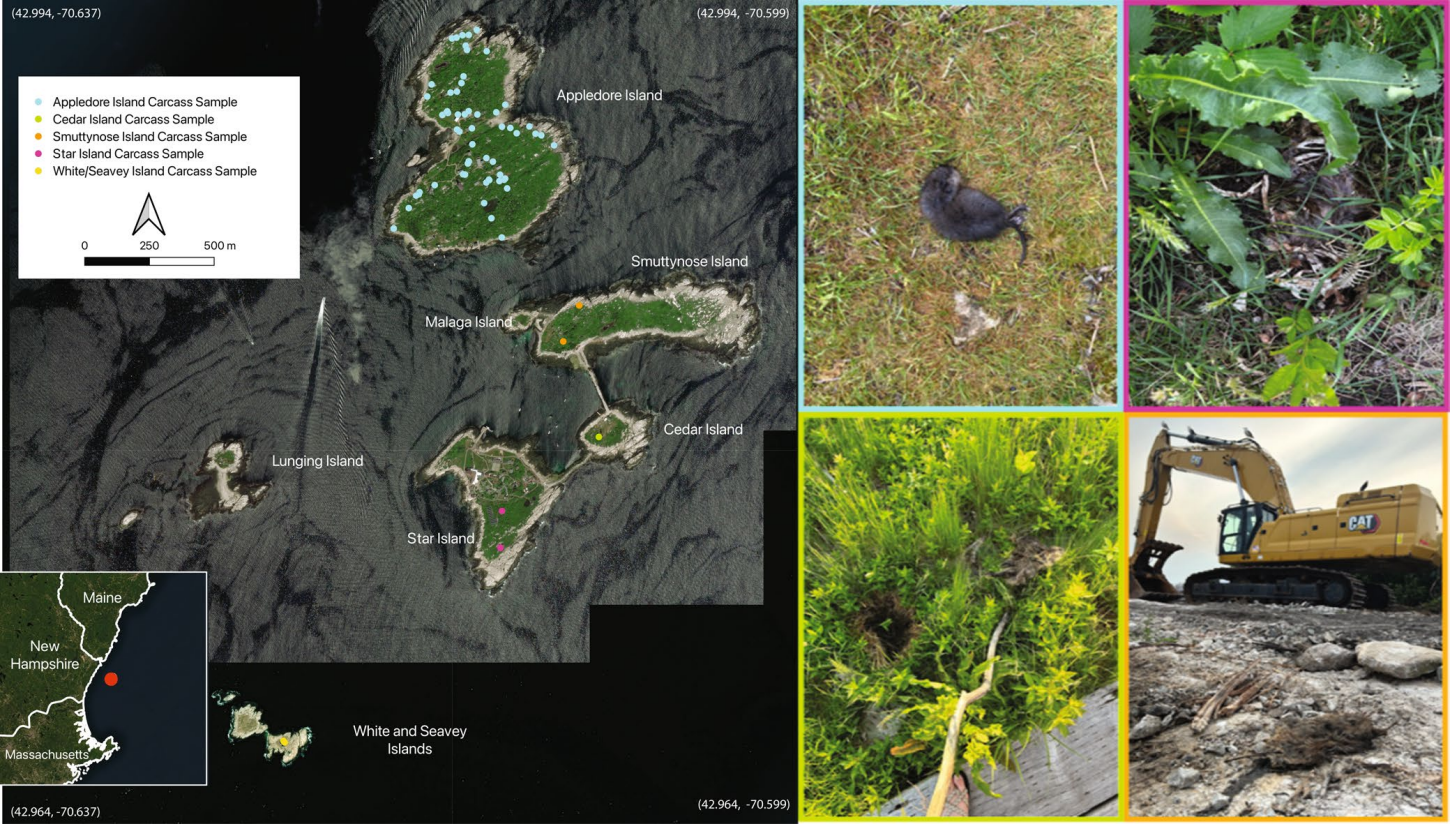
Map of the Isles of Shoals with muskrat occurrences based on whole/partial carcasses, with examples from each island shown in the images on the right (border colors correspond to color points on the map). The inset map shows the location of the archipelago relative to the eastern North American coastline and the states of Maine (ME), New Hampshire (NH), and Massachusetts (MA). Samples were collected from all islands except Malaga, Lunging, and Duck (not pictured). Samples were collected opportunistically on all islands, leading to a bias toward Appledore Island where the Shoals Marine Laboratory is based. Basemap (EPSG: 26919) data comes from ESRI World Imagery and was modified for visualization by M Zeltsar.

Muskrats are native to much of continental North America, including the northeast coast (Figure 2), and naturally occur on some large continental shelf islands (e.g., Mount Desert Island, Crowell 1986). However, there are several islands where their presence likely represents a living legacy of the fur trade. Genetic evidence suggests that the muskrats of the Isles of Shoals were introduced in the early 20th century for fur harvest (Mychajliw and Harrison 2014). The Isles of Shoals represents a different environment than muskrats typically experience, particularly as freshwater areas are restricted and ephemeral, and the island habitat largely consists of exposed granite intertidal surfaces, highly modified upland fields with seasonal human activity, and coastal shrub and grasslands (Eastwood et al. 2009; Kingsbury 1991; Nichols and Nichols 2008). Drawing from island biogeographic theory, insular muskrats should exhibit an expanded niche and high population densities (Lomolino 1984). Although anecdotes of the Isles of Shoals muskrats have been shared for decades among the island community, virtually nothing has been formally published (see Lyman 1988; Mychajliw and Harrison 2014) or recorded in community science databases (e.g., iNaturalist). Here, we report for the first time in the literature on the unique ecology of these insular muskrats, including their association with human structures and *Rattus* rats, upland habitat usage, and mortality from seabirds. The IUCN Red List currently does not recognize the existence of this insular population (Cassola 2016), and it is not actively managed by Maine or New Hampshire. Given ongoing reports of population declines across their North American range (Ahlers and Heske 2017; Sadowski and Bowman 2021; Ward et al. 2021), documenting the persistence of the Isles of Shoals muskrats may yield insight into the management of both native and invasive populations.

## 2 Methods

### 2.1 Study Area

The Isles of Shoals is located within the Gulf of Maine (centered at approximately 42°58′50″ N 70°36′54.5″ W), roughly 10 km from the nearest point of North American coastline. This group of small islands and ledges totals ~82 ha of land, ranging from 40 ha at the largest (Appledore) to 0.77 ha at the smallest (Malaga) (Figure 2), and they are separated by water depths of 18–30 m. Jurisdiction of the archipelago is split between Maine (Appledore, Cedar), New Hampshire (Star, White, Seavey, Lunging), and the USFWS National Wildlife Refuge system (Duck, Smuttynose, Malaga). Wabanaki people have been using islands in the Gulf of Maine for millennia (Sanger 1996), with subsequent colonial histories on the Isles of Shoals beginning in the 17th century linked to seasonal cod fishing that led to an established colony of several hundred people into the 18th century, and seasonal use for popular hotels in the 19th century (Kelso and Harrington 1989). Star, Cedar, Smuttynose, and Malaga have been connected by breakwaters since 1820 CE. Although we conducted surveys across the Isles of Shoals archipelago, we focused our observations on Appledore Island, which is the location of the Shoals Marine Laboratory (SML). SML is operated jointly by Cornell University and the University of New Hampshire and was built in 1966 following military usage of the island during WWII.

The Isles consist of aerially exposed igneous and metamorphic rock, forming jagged cliffs and glacially eroded “whalebacks” (Kingsbury 1991; Fowler‐Billings 1977), with disturbance regimes linked to nor'easters, hurricanes, and other wave‐altering events (Boden 1977). All islands have maritime rocky barrens and meadow natural communities (with the exception of Cedar Island) (Nichols and Nichols 2008). Appledore and Smuttynose contain the greatest variety of natural communities, including brackish water pools, coastal salt ponds, and shallow emergent marshes, coastal shoreline swales, highbush blueberry–winterberry shrub thickets, maritime intertidal rocky shores, and short graminoid–forb emergent mud flats (Nichols and Nichols 2008). In addition to the shallow emergent marshes, the only other habitat that would reasonably approximate mainland conditions on the Isles is the “Old Reservoir” on Appledore, which is a fenny marsh with *Sphagnum* cover, floating herb mats, cattails (*Typha*), duckweed (*Lemna*), rushes, irises, and sedges.

The Isles of Shoals lacks the typical suite of predators that muskrats would encounter in mainland ecosystems, most notably mammalian carnivores including minks ( *Neovison vison* ), red foxes ( *Vulpes vulpes* ), river otters ( *Lontra canadensis* ) and coyotes ( *Canis latrans* ). Less commonly, muskrats are predated by raptors such as owls, hawks, and ospreys. On the Isles, owls are occasionally present; instead, a potential predator unique to this archipelago is the American Herring Gull ( *Larus smithsonianus* ) and Great Black‐backed Gull ( *L. marinus* ), with hundreds of nests across several colonies during the summer breeding season (Covino et al. 2023).

The only formal scientific studies of muskrats on the Isles of Shoals have been conducted on Appledore (Lyman 1988; Mychajliw and Harrison 2014). A mark‐recapture study conducted in the 1980s yielded an estimate of ~27 muskrats/ha, which is substantially higher than mainland population densities (Lyman 1988). Genetic estimates calculated from sampling conducted in 2011 yielded an effective population size estimate of ~700–1200 individuals (Mychajliw and Harrison 2014). The absence of native nonvolant mammals aligns with the geographic and geologic qualities of the archipelago (Crowell 1986), and the only other mammals present on the island are due to past human‐mediated translocations (i.e., rats, *Rattus norvegicus* ).

### 2.2 Visual Surveys

**FIGURE 3:**
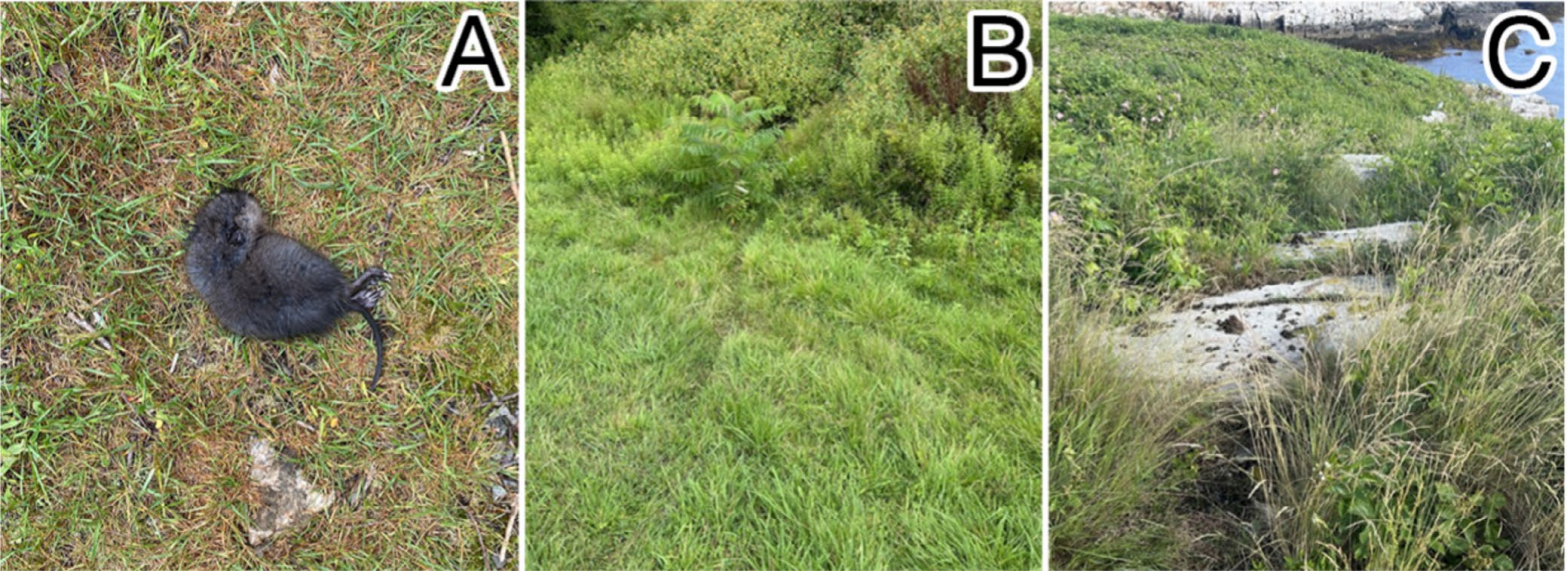
Photos of the different classifications of muskrat activity used during visual surveys. These classifications include direct observations of living or dead muskrats (A), runways created by muskrat movement (B), and individual scats and communal latrines (C). Photos by M Zeltsar and AM Mychajliw.

From June to August 2024, we conducted the first comprehensive survey of the Isles of Shoals in search of muskrat activity across all islands except Duck (which contains unexploded ordnances and is an active seal colony). We established the presence of muskrats for a given island based on observations of (a) live or dead individuals; (b) scat and latrines; and (c) ‘runways’, houses, and burrows in the vegetation (Figures 2 and 3).

We also visually surveyed Appledore Island more intensively relative to the other islands during this same period by systematically covering the entirety of the island's trail system, the entire perimeter of the coastline (excluding private property), all wetland areas on the island, and any other non‐forested or scrub‐brushed area across the island. These surveys determined the presence and absence of muskrats across Appledore Island based on observations of live muskrats, lodging and burrowing, scat, and tracks and/or runways (Figures 2 and 3).

Lastly, we announced our muskrat research to the over 150 students, staff, and faculty at SML, who subsequently helped report muskrat sightings and behaviors to augment our surveys (as in Gerraty et al. 2024). The community is highly engaged in documenting Appledore's marine and avian biodiversity (e.g., eBird), but terrestrial mammals have attracted less systematic attention.

### 2.3 Camera Trap Monitoring of Appledore Island

**FIGURE 4:**
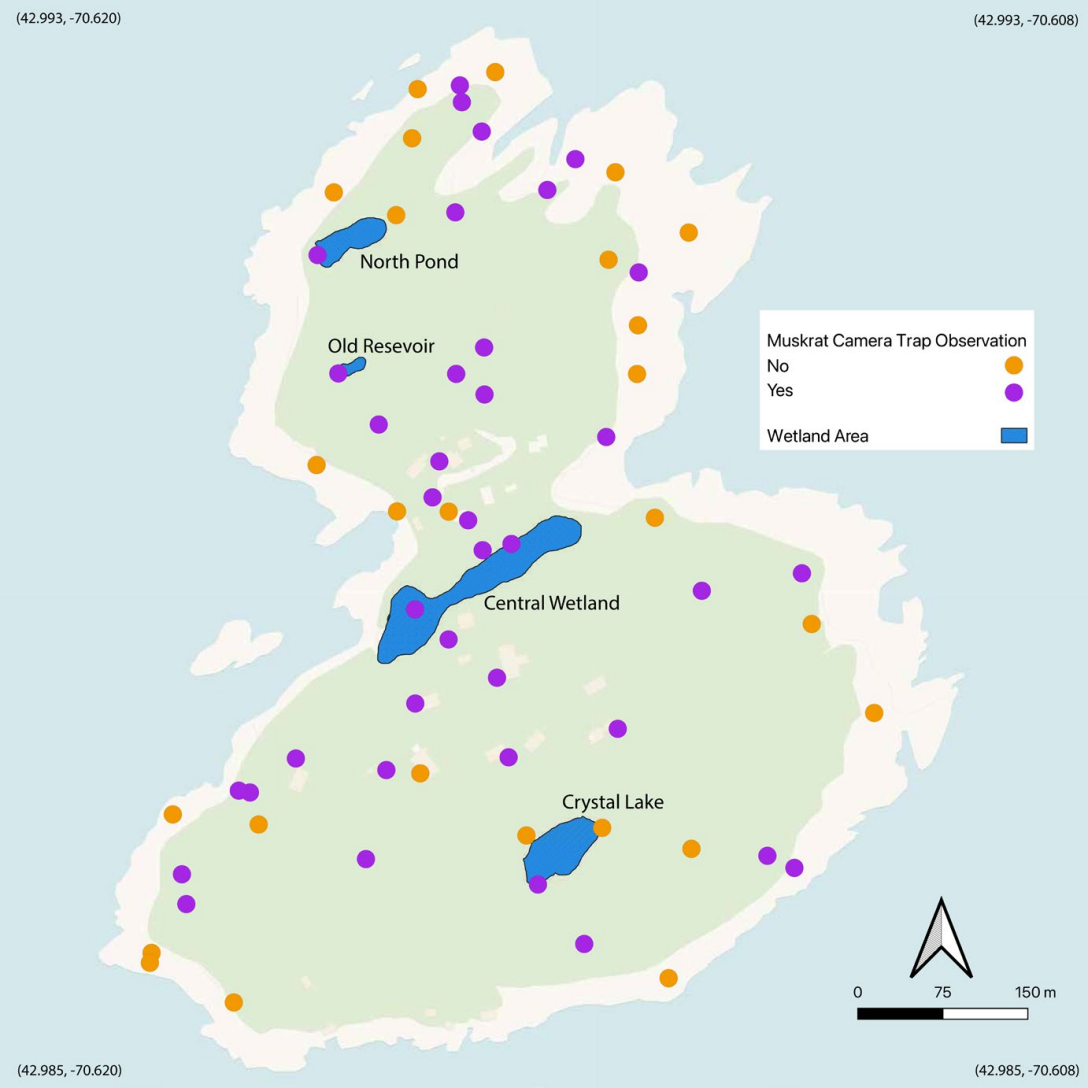
Map of presence/absence of muskrats as recorded by 64 camera traps on Appledore Island from June to August 2024. Presence is indicated in purple while absence is shown in orange. Basemap (EPSG: 26919) data from Voyager.

We deployed a total of 64 camera traps (Bushnell Trophy Cam HD) intermittently during June–August 2024, using a stratified sampling scheme based on ground type, soil texture, vegetation cover, gull presence, and proximity to human development. Camera settings followed the protocols of SNAPSHOT USA (Shamon et al. 2024) (Figure 4) and cameras were deployed for 2‐week rotational periods, which could have theoretically yielded a maximum of 3584 trap nights (8 weeks of 64 active cameras). Due to the high activity of gulls, camera traps frequently ran out of SD card storage space and/or battery power and thus were only partially functional across the designated 2‐week periods, complicating our ability to calculate trap nights across the full dataset equally and meaningfully. However, all camera traps were active and fully functional for at least 15 ‘trap nights’ each. We thus have a conservative total of 960 trap nights of camera data across the full array of 64 cameras.

## 3 Results

### 3.1 Distribution Across the Isles of Shoals and on Appledore Island

We confirmed the distribution of muskrats across all islands surveyed in the archipelago based on the occurrence of live animals, carcasses, tracks, actively used “runways”, and/or fresh feces (“latrines”) (Figure 2). We also confirmed that Appledore, Star, White, and Seavey islands have breeding populations, as evidenced by the presence of pregnant females, females accompanied by young, and/or neonates. We encountered a total of 62 whole and partial carcasses as well as isolated skeletal elements and clumps of hair as part of our research on Appledore Island.

Out of the total of 64 camera traps spread across Appledore Island, muskrats were captured (present) at 38 of the camera traps and not captured (absent) at 26 of the camera traps (Figure 4). With some exceptions, camera trap detection of muskrats shows a tendency to avoid areas with the highest concentration of gulls, which are often rocky areas with high exposure. Muskrats are widely distributed across the island and do not appear to cluster preferentially within freshwater areas such as Crystal Lake, Central Wetland, or the Old Reservoir (Figure 4). Instead, the majority of muskrat sightings are in areas of upland habitat or near human structures/modified areas, such as buildings, and they are commonly encountered in open grass fields. Muskrat fecal towers (“latrines”) can be found on granite ledges on the periphery of vegetation despite the presence of nesting gulls. In these areas, small brackish water pools form within the trap dikes leading to the intertidal zone and accumulate enough soil for small amounts of vegetation to grow (Kingsbury 1991). These pools contain a three trophic level food web that muskrats may exploit for freshwater and/or nutrients from the chlorophyte algae (Pellowe‐Wagstaff and Simonis 2014). However, our cameras that were positioned along these granite ledges did not consistently detect muskrats despite the presence of their feces, which could be due to the high gull activity that frequently filled camera memory cards and exhausted camera batteries.

### 3.2 Burrow and Lodge Construction

**FIGURE 5:**
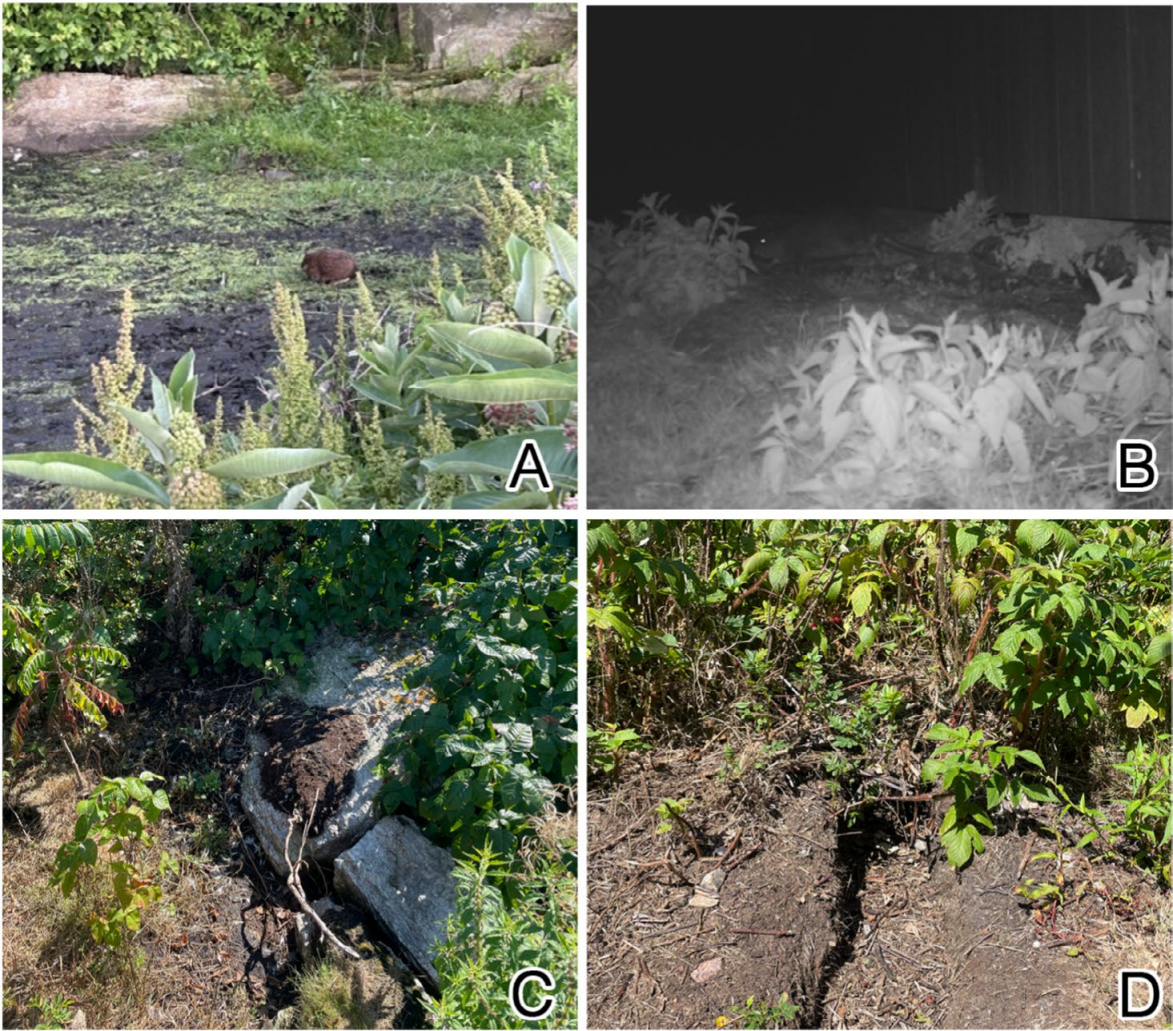
Images of muskrat lodging environments and behavior on Appledore Island. (A) Excavating and mounding dirt on top of rocky surfaces, (B) Utilizing space underneath buildings for housing and shelter, (C) building stick lodges on dry land underneath scrub brush, (D) burrowing into dry soil. All photos by M Zeltsar and AM Mychajliw.

Muskrats create a variety of structures on Appledore—some typical of mainland behaviors and some atypical. In general, the location and structure of a muskrat house is determined by soil type, nearby emergent vegetation, slope of the bank, and water depth (Willner 1980; Nadeau et al. 1995). Despite the presence of some freshwater and marsh areas (e.g., Crystal Lake), muskrats do not appear to create typical “floating” lodges or “push‐ups” within bodies of water. Instead, muskrats on Appledore more generally create lodges on dry land within brush, connected to burrows and tunnels in soft soils/mud (Figure 5). They also utilize existing human structures such as wooden pallets and the spaces under buildings to create modified burrows (Figure 5).

### 3.3 Species Interactions

**FIGURE 6:**
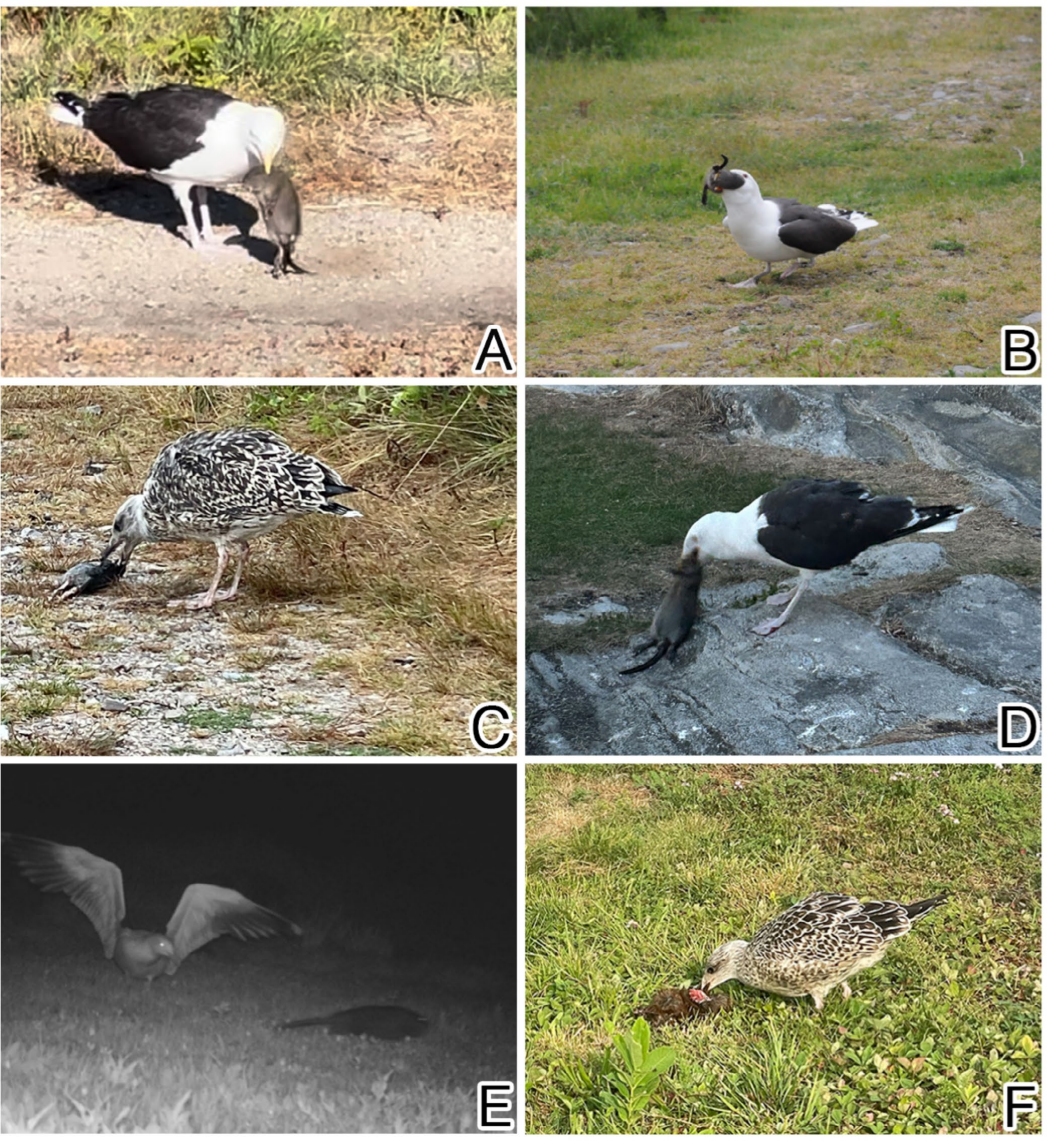
Images of gulls predating, harassing, and scavenging rats and muskrats on Appledore Island. (A, credit: J. Jerome) adult Great Black‐backed Gull actively killing an adult muskrat, (B, credit: J. Stilwell) adult Great Black‐backed Gull actively killing and consuming a juvenile muskrat, (C, credit: M. Zeltsar) Juvenile Great Black‐Backed Gull predating a Norway rat, (D, credit: M. Ponce) adult Great Black‐Backed Gull scavenging a muskrat, (E, credit M. Zeltsar) Adult Great Black‐Backed Gull chasing and harassing an adult muskrat, (F, credit M. Zeltsar) juvenile American Herring Gull scavenging a dead muskrat.

Based on camera trap observations and the location of feces, it is clear that muskrats co‐occur with both Herring Gulls and Great Black‐backed Gulls, and they are regularly seen foraging in areas near active nests with chicks. Muskrats are frequently the object of gull territorial aggression during the nesting season, and we have observed muskrat injury and mortality as a result of defensive behaviors around nests and chicks of both species. Additionally, we have also observed instances of both carcass scavenging and active predation attempts by Great Black‐backed Gulls, the larger and more aggressive of the two species (Figure 6); the Great Black‐backed Gull can reach up to 2000 g (Good 1998), surpassing the size of most juvenile muskrats. Isolated muskrat skeletal elements are often encountered near gull breeding sites. Predation by Barred Owls ( *Strix varia* ) has also been observed, and small muskrat skeletal elements have been recovered from regurgitant. No other predators appear to be active on the archipelago.

**FIGURE 7:**
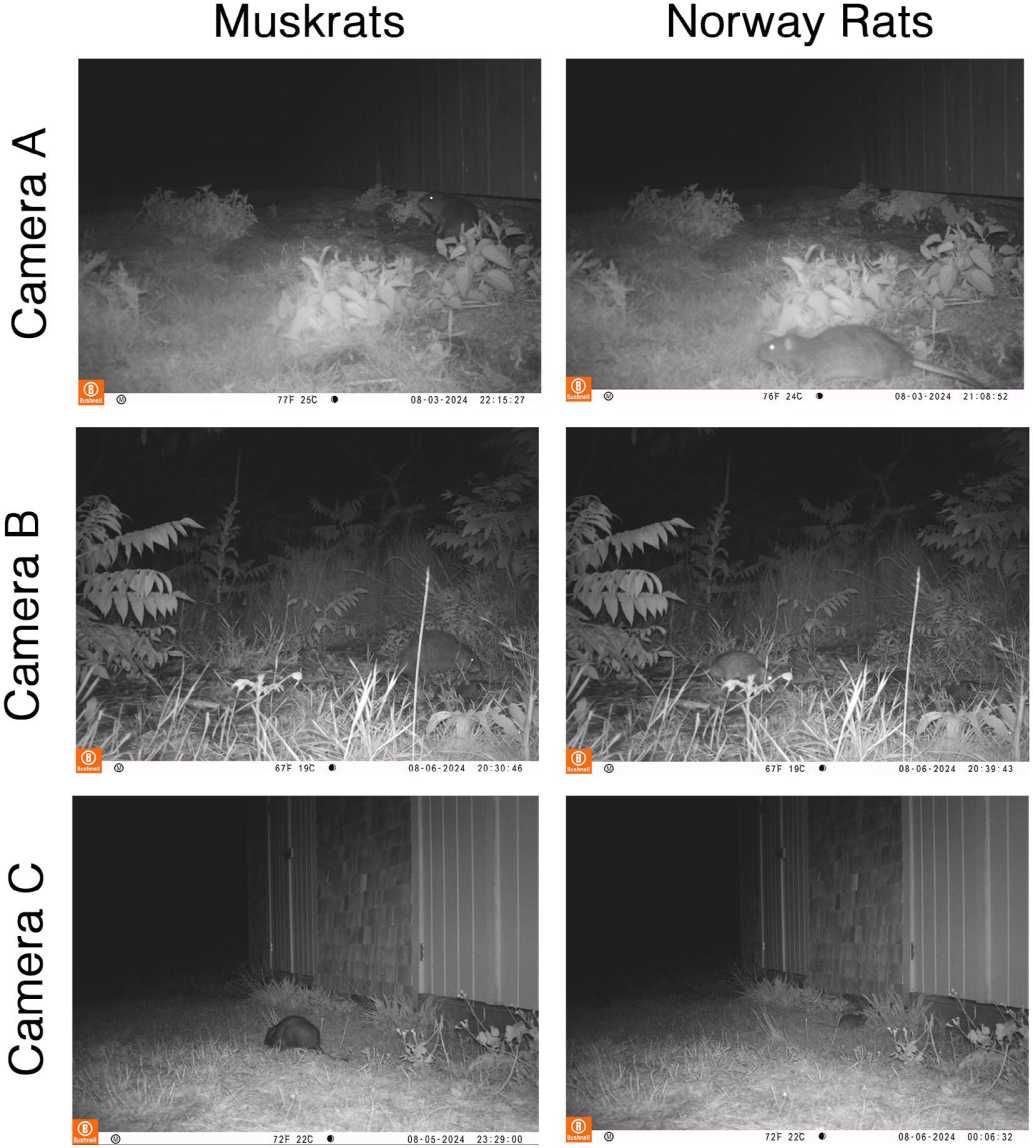
Images of muskrats and rat coexistence. Camera trap sightings of muskrats and rats co‐occurring near human development areas on Appledore Island. All three sets of photos were taken on the same night within 90 min of each other.

**FIGURE 8:**
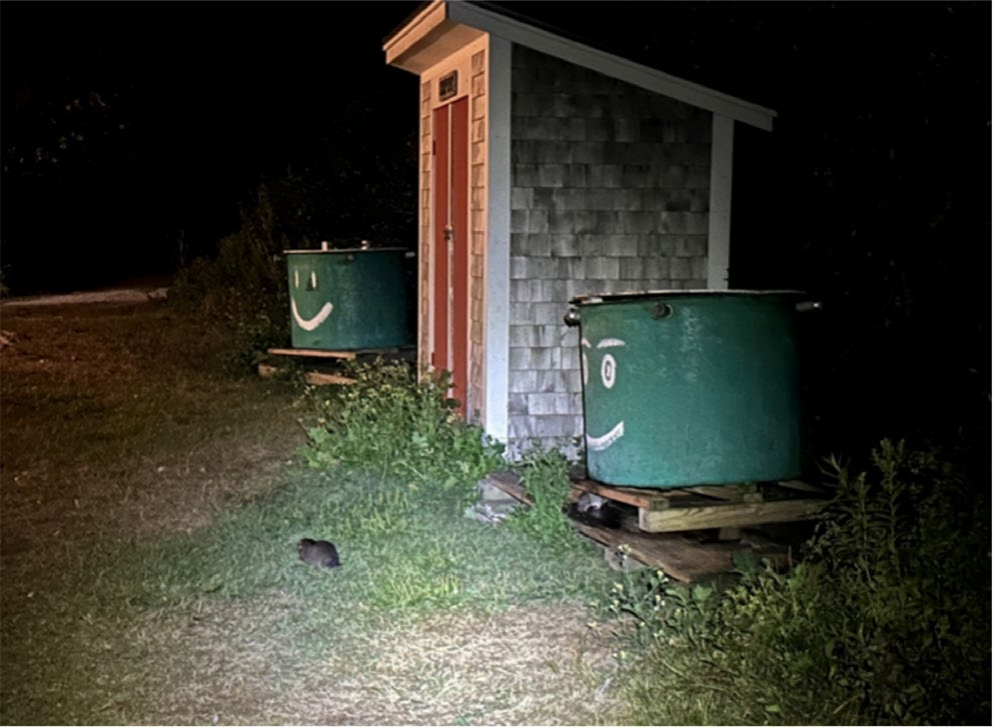
Image of juvenile muskrat and brown rat foraging simultaneously in a grass field associated with compost bins. Photo by AM Mychajliw.

**FIGURE 9:**
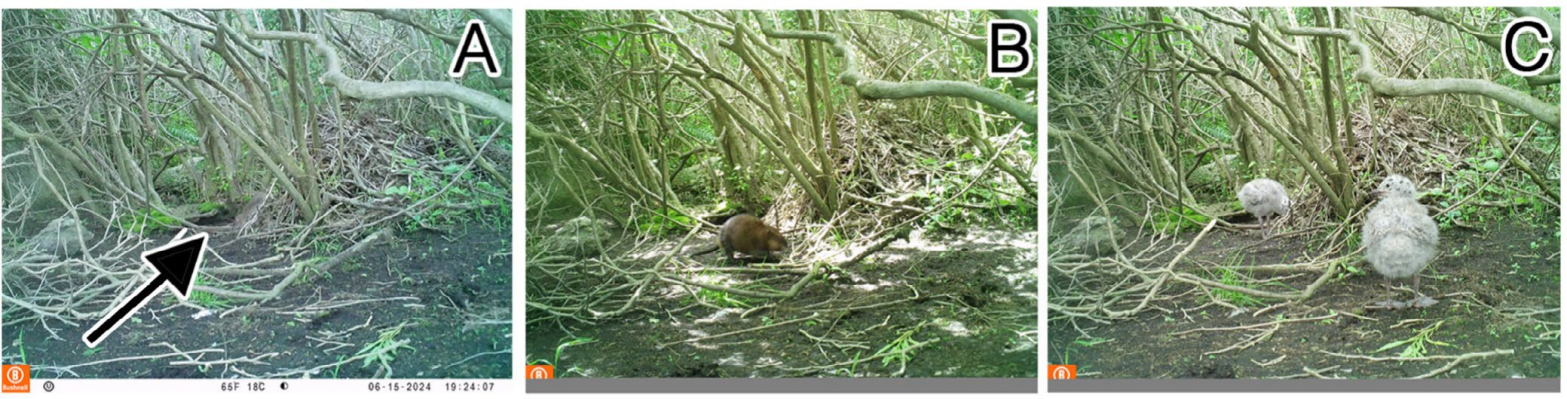
A photographic example from camera traps of the overlap and cooccurrence between rats (A), muskrats (B), and gulls (C) on Appledore Island.

We documented the frequent co‐occurrence of muskrats and Norway rats ( *Rattus norvegicus* ), the only two common terrestrial mammals on Appledore Island (Figure 7). Although no direct interactions were observed, they have been documented foraging concurrently in human‐modified areas, such as compost and garbage bins, storage sheds, and campus buildings (Figure 8) as well as near terrestrial lodges (Figure 9).

## 4 Discussion

Muskrats were widely translocated outside of North America to facilitate fur production in the early–mid 20th century, resulting in invasive populations across Europe, Japan, Korea, Russia, and Argentina, among other areas, often in tandem with their cousins, the North American beaver ( *Castor canadensis* ) (Cassola 2016; Crego et al. 2016; Nentwig et al. 2010). Because they are considered pests, research in recent decades has focused mostly on their invasive range. This emerging body of literature suggests that, despite being introduced across multiple biogeographic barriers, invasive muskrats appear to have conserved their continental North American niche and are restricted to wetland habitats (e.g., Ruys et al. 2011; Skyrienė and Paulauskas 2012; Nummi et al. 2006; Kim et al. 2020).

In contrast to the abovementioned studies, muskrats on the Isles of Shoals appear to diverge from their obligate association with wetlands for food and shelter. The presence of water has been considered the greatest selection pressure affecting muskrats, striking a balance between being deep enough to maintain travel routes and underwater access to lodges, but shallow enough to still have the desired emergent vegetation food items and structural vegetation diversity (Ahlers et al. 2015; Bellrose and Brown 1941; Errington et al. 1963; Proulx and Gilbert 1984; Virgl and Messier 1996; Ward and Gorelick 2018). Appledore Island does have freshwater wetland and marshes, yet these do not appear to be the most commonly used spaces on the island. Muskrats on Appledore do not appear to create lodges or push‐ups with cattails or other emergent vegetation but instead rely solely on terrestrial structures and burrows. In mainland populations, muskrats only shift toward the use of burrows rather than water lodges only when suitable building materials are unavailable or when water depths are too low (< 15 cm; Proulx and Gilbert 1984). Thus, it is likely that other factors are allowing Shoals muskrats to create structures outside of wetlands.

Predation on islands may be a key factor in shaping whether muskrats expand their niche, aligning with expectations from island biogeography. No deviations from typical wetland behaviors have been reported from native insular populations of muskrats on Prince Edward Island, which also has a full suite of continental mammal fauna including carnivores (Sobey 2007). Conversely, on Newfoundland Island, muskrats are considered native, though the island lacks their preferred forms of emergent vegetation (e.g., cattails), and therefore muskrats rely on bank burrows rather than traditional lodges (Soper and Payne 1997). This marginal habitat makes them more vulnerable to predation by minks, which were introduced as a result of fur farms in the 1930s (Soper and Payne 1997; Northcott et al. 1974). The expanded spatial distribution of muskrats into marginal upland habitats on Appledore may reflect a combination of territoriality during the breeding season and relaxed predation in the absence of mammalian carnivores. In mainland populations, muskrats become aggressive during the summer breeding season and maintain dominance hierarchies that push low‐ranking muskrats to suboptimal sites (Proulx and Gilbert 1984). Some areas on Appledore could be occupied by displaced low‐ranking individuals and juveniles (Skinner and Skinner 2008; Marinelli and Messier 1993). Use of the freshwater rock pools on the exposed perimeter of the island (as evidenced by the presence of scat and latrines creating paths from the edge of vegetation to the pools) could be a result of low‐ranking individuals getting pushed to marginal freshwater sources. There are an estimated 1500 of such freshwater rock pools on the island above the high tide line (Pellowe‐Wagstaff and Simonis 2014), and these pools are often surrounded by aggressive nesting seabirds during the summer breeding season.

The only similar context to the Isles of Shoals reported in the literature is a historically introduced population on another Maine island without native mammalian carnivores (Clough 1987). Damariscove Island is an 85 ha island located 4.2 km from the mainland (for comparison, Appledore is about half the size and double the distance away, 40 ha and 10 km, respectively). Muskrats were introduced to Damariscove in the 1940s and have been actively trapped since the 1970s. Similar to Appledore, the vegetation on Damariscove is also mostly coastal shrub and grassland with a paucity of freshwater or wetland areas that disappear by the summer. There, muskrats live in underground burrows, use above ground runways, and eat upland vegetation, consistent with observations of continental muskrat populations experiencing demographic decline and marginal conditions (Errington et al. 1963); indeed, Clough (1987) observes that “the Damariscove muskrats subsist regularly on what would be an emergency diet elsewhere”. These muskrats exhibit a tension of island life: on the one hand, the poor quality food and habitat have led to smaller litter sizes, but on the other hand, there is lower predation pressure (due to the lack of continental mammal fauna) and higher overall survival (Clough 1987).

Based on our observations, and the known smaller litter size of Appledore muskrats (Lyman 1988), we propose that Appledore is also likely a marginal habitat that has pushed muskrats to the edge of their flexibility, but the lack of continental mammalian predators allows them to persist at higher population densities than mainland populations (Lyman 1988). Muskrat populations are known to experience significant demographic fluctuations and mortality events (Erb et al. 2000), particularly in the winter, which could result in boom‐bust cycles on the island. For example, Errington et al. (1963) describes a particularly stressed population in which “dead muskrats, with bodies either intact or more or less consumed by flesh eaters, were found in many places within a quarter‐mile of the marsh… the bones and foot of a leg would lie in one place, a patch of fur or a skull in another; and there were bodies frozen into the ice but with fleshy parts hollowed out by cannibalistic muskrats gnawing at them from above”. Unfortunately, no information is available about how winter conditions may affect muskrats on the Isles of Shoals as of yet, though there is semi‐regular snowy owl and other raptor activity (e.g., peregrine falcons).

Although continental mammalian predators are not present, birds appear to instead be a source of mortality during the summer. The most frequent cause of mortality we witnessed was linked to the diurnal activity of gulls (family Laridae, genus *Larus*). This mortality may represent a combination of territorial aggression as well as active predation events. The Isles of Shoals is a seasonal mixed colony breeding area for seabirds, including hundreds of nesting Great Black‐backed Gulls ( *L. marinus* ) and American Herring Gulls ( *Larus argentatus* ) (Ellis and Good 2006; Savoca et al. 2011). These gulls are highly territorial and protective of their nesting sites during the breeding season, resulting in frequent intra‐ and interspecific aggression (Ellis and Good 2006). Reproductive success of the gulls varies widely across Appledore Island; tolerance to human disturbance and intra and interspecific competition for optimal nesting sites has resulted in both dense and loose colony contexts that shape the threat tolerance and aggression by gulls (Ellis and Good 2006; Savoca et al. 2011; Burger and Gochfeld 1983), which in turn could shape the spatial distribution of muskrats we witness here. For example, muskrats have been observed limping on the island with injuries to their hind limbs and potentially spinal cords, which would be consistent with gull strikes to muskrats fleeing from a nest site. Additionally, we have documented some cases of predation by Barred Owls ( *Strix varia* ) on camera traps and through the presence of bones in regurgitant—which would be expected based on interactions in mainland contexts in Maine (Mendall 1944).

In the Gulf of Maine, Herring Gulls and Great Black‐backed Gulls are both considered generalist predators with a diet consisting mostly of fish and crabs, but Great Black‐backed Gulls are larger (up to double the size; 800–980 g vs. 1300–2000 g), more aggressive, and feed at higher trophic positions than Herring Gulls (Good 1998; Ellis and Good 2006). Great Black‐backed Gulls in the UK have been documented to regularly consume mammals, mostly European rabbits ( *Oryctolagus cuniculus* ) (Westerberg et al. 2019). In the Netherlands, European Herring Gulls ( *Larus argentatus* ) and Lesser Black–backed Gulls ( *Larus fuscus* ) are known to consume a wider range of mammalian prey encountered in human‐modified environments (farm fields, roadsides), including western hedgehogs ( *Erinaceus europaeus* ), shrews (Soricidae), voles (Cricetidae: Arvicolinae), mice (Muridae), moles ( *Talpa europaea* ), brown rats ( *Rattus norvegicus* ), European rabbits, and common brown hares ( *Lepus europaeus* ) (Camphuysen et al. 2010). Here we report the first visually documented cases of predation—not just scavenging or territorial defense—of gulls on muskrats. Consistent with the known ecology of the two gull species present, such behaviors were only observed for the larger, more aggressive species, the Great Black‐backed Gull. Although adult muskrats would be too physically large to serve as viable prey for these species, the coincidence of the breeding season of both gulls and muskrats on Appledore facilitates the availability of juvenile muskrats as appropriate‐sized prey. Loss of antipredator behavior and decreased vigilance would also align with studies of insular species (Blumstein and Daniel 2005).

This type of unlikely interaction is increasingly common in the Anthropocene, with novelty in the predation sequence resulting from changes in species overlap and changes in the “stage” or environment in which predation occurs: new actors co‐occurring in new places or as a result of sequentially shifted baselines (Guiden et al. 2019). In this case, the pathways to the intersection of gulls and muskrats are a result of a complicated context and series of contingent events in the Gulf of Maine. Muskrats, as noted, were introduced to the Isles of Shoals in the early–mid 1900s (Mychajliw and Harrison 2014), likely close to the timing of WWII. Conversely, baselines have been shifting for gulls in the Gulf of Maine for centuries: after persecution led to significant declines in the late 1800s and early 1900s, populations of Herring Gulls peaked in the 1970s as a result of increased protection and access to landfills, and have been declining with concurrent increases in Great Black‐backed Gulls and covering of landfills (Taylor et al. 2024). At the time of initial muskrat introduction to the Isles of Shoals, the gull population would have been much lower than it is today, potentially providing an opportunity for initial population growth and expansion on the island.

We report another unexpected species interaction for the Isles of Shoals: that of muskrats and invasive brown rats sharing human‐dominated spaces on Appledore. The literature only documents three previous cases of interaction of these species, with rats seeking shelter in muskrat lodges within the muskrat native range (Kiviat 1978; de Szalay and Cassidy 2001). Muskrats on Appledore share some of the qualities associated with anthrodependent taxa, and flexibility in territoriality, feeding behavior, and group foraging may allow them to live at higher population densities in human‐dominated spaces with fluctuating resources and modified environments (Hulme‐Beaman et al. 2016). Additional research is necessary to determine whether muskrats and rats have a commensal, facultative, or competitive relationship.

The information reported here illuminates a previously unknown potential for muskrat behavior and ecology, particularly regarding the conditions that support their use of marginal habitat and ability to interact with novel predators and competitors. This adds a unique perspective to a growing paradox of native range decline coupled with non‐native range expansion (Hong et al. 2024). Recent studies suggest that muskrat populations in North America have been declining over the past two decades within their native range, as reported by pelt sales and resurveys of historical populations (Ahlers and Heske 2017; Roberts and Crimmins 2010; Sadowski and Bowman 2021). The intersecting effects of anthropogenic climate change, invasive plant species, and habitat alteration are potential causes of these declines (Turner et al. 2019; Ward et al. 2020; Melvin 2024). Yet, the Isles of Shoals muskrats appear to contend with many similar challenges and are flexible in their vegetation and water use, providing a glimpse of hope for persistence in Anthropocene conditions.

Muskrats have multiple dimensions of importance to people, as evidenced by their widely human‐augmented range across hemispheres. The species was the most commonly harvested wild furbearer in North America of the 20th century and is still among the most commonly trapped mammals in North America (Obbard et al. 1987; Cassola 2016). Zooarchaeological records document the harvest of muskrats for millennia in New England ecosystems (Mychajliw et al. 2023), and this species remains important to Indigenous communities across the continent; muskrats are both ecological indicators and symbols of cultural resilience (Straka et al. 2018; Turner 2018). In Maine, muskrats—known as “kiwhos” in Passamaquoddy and “ki'kwesu” in Mi'kmaq—continue to be in relationship with Wabanaki communities and are associated with the traditional medicinal muskrat root ( *Acorus calamus* ; Baumflek et al. 2015). The evolving relationship between humans and muskrats on the Isles of Shoals contributes to a richer picture of shifting ecological and cultural baselines for furbearing mammals and presents new collaborative research opportunities to expand perspectives on their management.

## Author Contributions

**Alexis M. Mychajliw:** conceptualization (lead), data curation (supporting), funding acquisition (lead), investigation (equal), methodology (equal), resources (lead), visualization (supporting), writing – original draft (lead), writing – review and editing (lead). **Max Zeltsar:** conceptualization (equal), data curation (lead), formal analysis (equal), funding acquisition (supporting), investigation (equal), methodology (equal), resources (equal), software (lead), visualization (lead), writing – original draft (supporting), writing – review and editing (equal). **John Dennis:** conceptualization (equal), investigation (supporting), methodology (supporting), writing – review and editing (equal). **Maddie E. Ellms:** investigation (supporting), resources (supporting), visualization (supporting), writing – review and editing (equal). **Dylan Titmuss:** resources (supporting). **Kristen M. Covino:** resources (supporting), writing – review and editing (lead). **Sara Williams:** investigation (supporting), methodology (supporting). **Shrushti Modi:** investigation (supporting), methodology (supporting). **Courtney A. Hofman:** conceptualization (supporting), funding acquisition (equal), project administration (equal), resources (equal), writing – review and editing (equal).

## Conflicts of Interest

The authors declare no conflicts of interest.

## Data Availability

All relevant data are present within the manuscript in the form of maps and images.
